# Case Report: Durable remission after abscopal effect following transcatheter hepatic arterial embolization in a patient with mucosal melanoma refractory to immunotherapy

**DOI:** 10.3389/fimmu.2025.1565355

**Published:** 2025-04-10

**Authors:** Lynsey M. Claus, Hannah E. Kostan, Marshall E. Hicks, Rony Avritscher, Michael A. Davies

**Affiliations:** ^1^ Department of Interventional Radiology, The University of Texas MD Anderson Cancer Center, Houston, TX, United States; ^2^ Department of Interventional Radiology, Baylor College of Medicine, Houston, TX, United States; ^3^ Department of Melanoma Medical Oncology, The University of Texas MD Anderson Cancer Center, Houston, TX, United States

**Keywords:** mucosal melanoma, abscopal, immunotherapy, checkpoint inhibitors, transarterial embolization

## Abstract

Mucosal melanoma, a rare subtype of melanoma affecting mucosal surfaces, presents significant challenges in diagnosis and treatment, particularly due to its low programmed death-ligand 1 (PD-L1) expression and reduced response to immune checkpoint inhibitors (ICIs). This case report describes a 58-year-old woman with metastatic nasal mucosal melanoma initially resistant to neoadjuvant ipilimumab and nivolumab. After undergoing hepatic transcatheter arterial embolization, she experienced an unexpected abscopal effect, where distant metastases showed near-complete resolution despite prior lack of response to immunotherapy. The patient’s disease initially progressed despite two cycles of ICI treatment, and further immunotherapy with nivolumab and relatimab did not improve her condition. Subsequently, after bland embolization of a dominant hepatic mass, the patient received re-challenged combination immunotherapy with ipilimumab and nivolumab, leading to significant regression in metastatic sites including the liver, lungs, lymph nodes, and bones. This response contrasts with prior reports and highlights a potential mechanism by which embolization therapies may alter the immune microenvironment and enhance the efficacy of immunotherapy. The abscopal effect observed following local hepatic embolization may be attributed to tumor-induced immune activation, which is further amplified by ICI treatment. This case underscores the potential for integrating local embolization with immunotherapy to overcome resistance in metastatic melanoma, particularly in mucosal subtypes. Further research is necessary to elucidate the immune mechanisms underlying these responses and to optimize treatment strategies for melanoma patients.

## Introduction

Melanoma, a skin cancer originating in melanocytes, is typically cutaneous but can, in rare instances, manifest as mucosal melanoma, affecting mucous membranes such as those in the nasal cavity, sinuses, anorectal and vulvovaginal regions, and less commonly in the gastrointestinal tract. While mucosal melanoma is uncommon among Caucasians, it accounts for up to 20%-30% of melanomas in Asian populations (1). This subtype often presents with subtle or absent early clinical signs, which can delay diagnosis and lead to poor patient prognosis. Mucosal melanomas are also known to show lower levels of programmed death-ligand 1 (PD-L1) expression in the tumor immune microenvironment (2). Despite melanoma’s strong immunogenicity, which supports the use of immune checkpoint inhibitors (ICIs), these therapies – designed to prevent immune evasion, facilitate recognition of tumor-specific neoantigens, and generate a systemic cell-mediated immune response- have shown reduced efficacy in mucosal melanoma (3, 4). This case report highlights a patient with metastatic nasal mucosal melanoma who experienced an abscopal effect following local bland embolization and combination immunotherapy with ipilimumab and nivolumab, after initially not responding to immunotherapy.

## Case description

A 58-year-old female (informed consent obtained) presented with a four-month history of nasal obstruction and intermittent bleeding. Imaging revealed a 4 cm mass in the right nasal cavity, from which endoscopic biopsies were taken. Pathology revealed melanoma fragments with areas of hemorrhage and minute fragments of respiratory epithelium. Tumor cells were positive for Mart 1 and focally for S100, but negative for cytokeratin (CK) 8/18, neuron-specific enolase (NSE), p63, CK7, and pan-CK (AE1/AE3). Rare cells also stained positive for epithelial membrane antigen (EMA). Molecular testing found a frameshift mutation in the *Nf1* gene, and amplification of *Myc* and *Nbn.* The overall profile confirmed the melanocytic origin of the tumor. Baseline imaging, including PET-CT, ultrasound of the neck, and MRI of the brain, found clinically localized disease, with no radiographic evidence of regional or distant metastases. Thus, she was diagnosed with T3bN0MX biopsy-proven right nasal mucosal melanoma.

**Figure 1:**
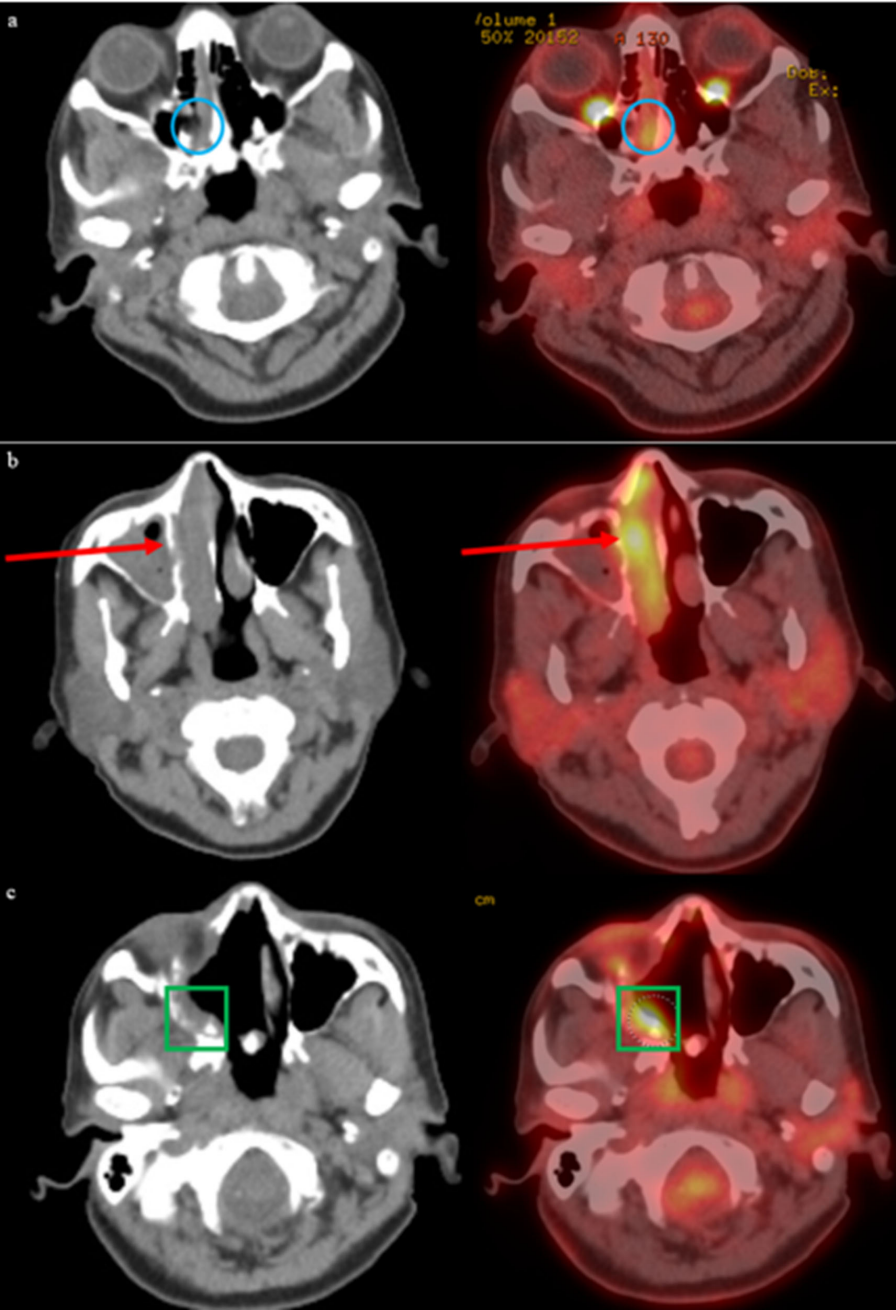
Progression of biopsy-confirmed melanoma in right nasal cavity is detected by initial imaging findings revealing T3 disease. Post-surgical changes in sinus and surrounding soft tissues is observed with PET-CT, with concern for residual disease. **(a)** Initial PET-CT on 8/26/2021 reveals a foci of asymmetric metabolism corresponding to the soft tissue abnormality in the right nasal cavity (blue circle). **(b)** Follow-up PET-CT 10/21/2021 revealed increasing soft tissue within the right nasal cavity with heterogeneous activity (red arrow). **(c)** Postoperative changes related to endoscopic nasal and skull base resection with associated post radiation changes seen on PET-CT dated 1/26/2022, with increased FDG-activity along the right posterior maxillary sinus and right ethmoidectomy bed (green square).

**Figure 2:**
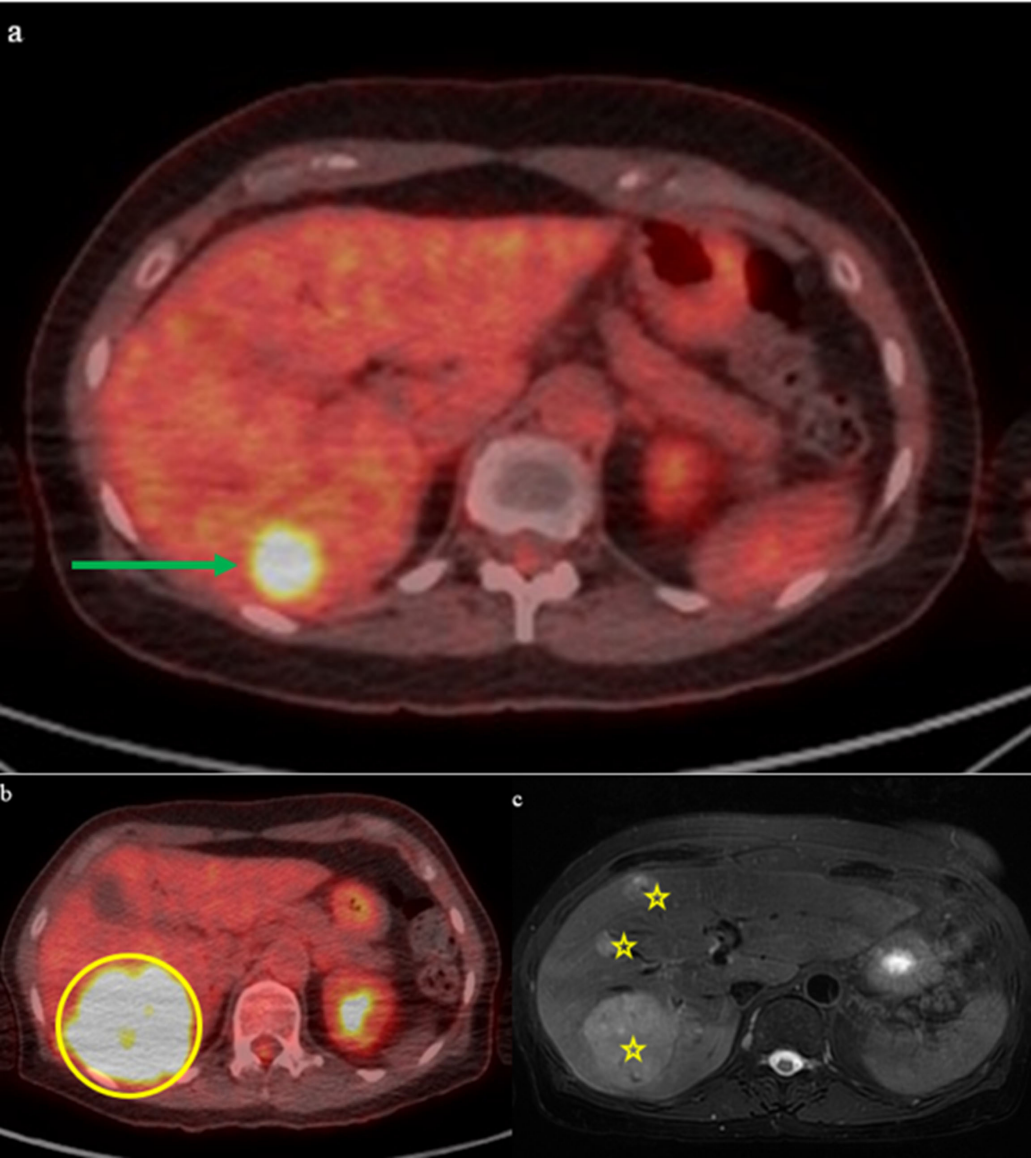
PET-CT scans identified new hypermetabolic nodes in the neck and a liver mass, both likely melanoma metastases. Follow-up PET-CT and MRI confirmed progression of hepatic metastases and raised suspicion of tumor foci in the abdominothoracic wall. **(a)** PET-CT on 8/31/2022 shows new mass in right lobe of liver measuring approximately 3 cm (green arrow). **(b)** Follow-up PET-CT in December 2022 shows interval progression of FDG-avid hepatic metastasis (yellow circle). **(c)** Associated follow-up MRI in December 2022 demonstrates multifocal hepatic metastatic disease progression (yellow stars).

**Figure 3:**
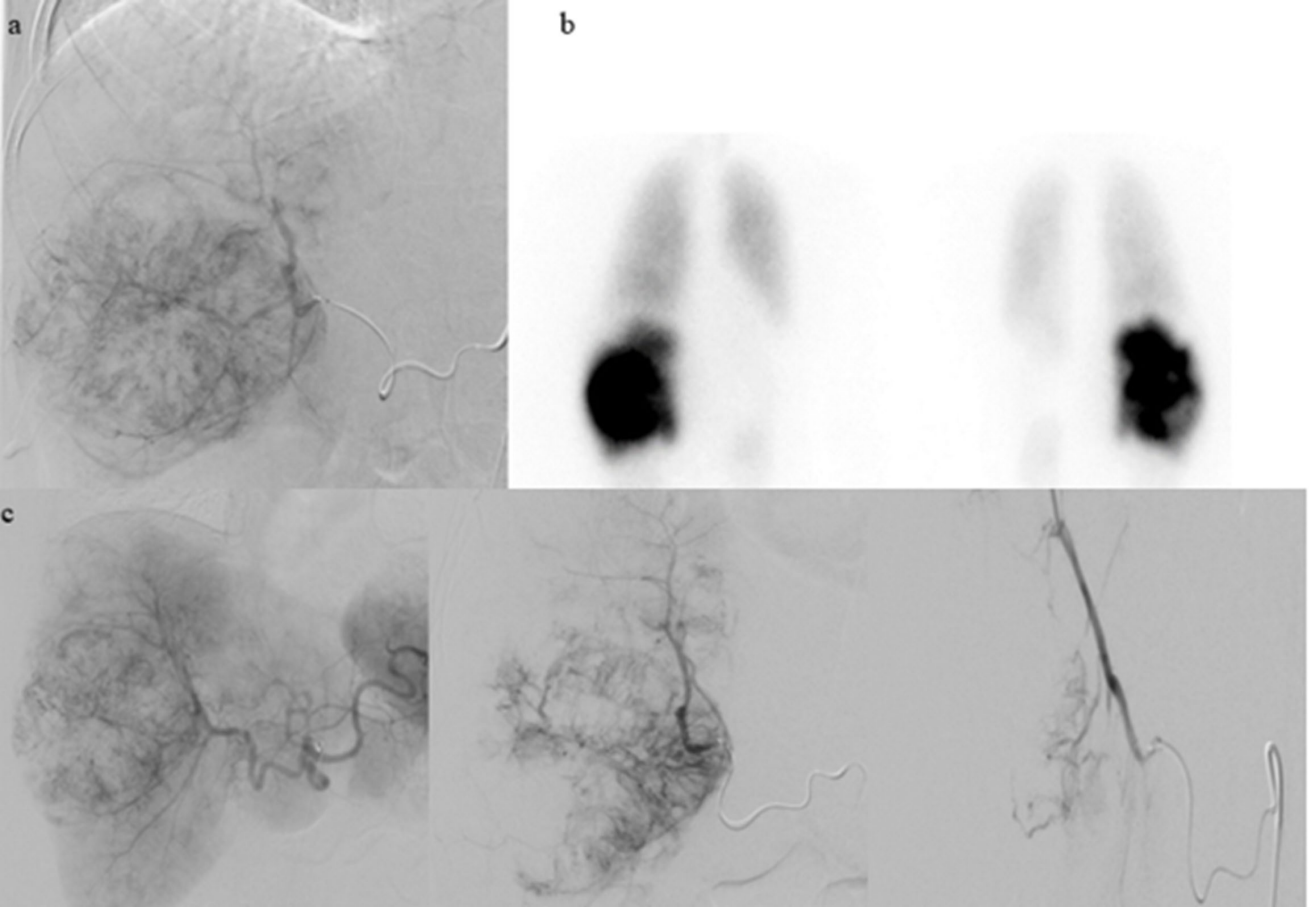
Due to the significant advancement of liver disease, the patient was referred to interventional radiology for Y90 TARE. Given the high lung shunt fraction, bland embolization with larger particles was chosen as the treatment approach. **(a)** Planning hepatic angiography on 1/13/2023 with Tc-99m injection performed. **(b)** NM lung shunt fraction determined to be 26.3%. **(c)** Successful transcatheter hepatic arterial embolization of the right hepatic mass performed on 2/2/2023 using Gelfoam slurry and particles.

The patient was initially treated with two cycles of neoadjuvant ipilimumab (1 mg/kg) and nivolumab (3 mg/kg). Restaging PET-CT after neoadjuvant therapy found increasing soft tissue mass within the right nasal cavity with near complete opacification of right paranasal sinuses and no evidence of metastatic disease. MRI of the face also showed interval growth of the nasal cavity tumor. The patient underwent endoscopic endonasal surgical resection of the nasal mass with ethmoidectomy and exploration of the frontal sinus, including removal of tissue from the sinus (**Figure 1**). Pathology review confirmed 3 cm mucosal melanoma extending to the tissue edges with 70% viable tumor, consistent with a pathological non-response to neoadjuvant immunotherapy (5). To reduce the risk of local recurrence, the patient subsequently received adjuvant radiation therapy with five cycles of 600 cGy to the nasal cavity, totaling 3000 cGy. Adjuvant chemotherapy was discussed with the patient, but she declined this treatment based on toxicity rates and limited data in the current therapeutic landscape of melanoma. The patient was followed with surveillance imaging at 3-month intervals. Approximately six months after completion of external beam radiation, a PET-CT scan revealed a new 1 cm Fluorodeoxyglucose (FDG)-avid right retropharyngeal lymph node and a new 2.8 cm FDG-avid lesion in the liver. The liver lesion was biopsied and confirmed metastatic melanoma. This finding led to re-initiation of immunotherapy, but this time with nivolumab and relatimab due to the lack of response to neoadjuvant ipilimumab and nivolumab. Restaging after 3 cycles of immunotherapy showed disease progression, with increased FDG avidity of the retropharyngeal lymph node, increased size (7.8 cm) of the biopsy-confirmed liver metastasis, and new liver lesions (**Figure 2**). Consequently, she was referred for liver-directed therapy for rapidly enlarging hepatic metastases. Although initially considered for Yttrium-90 (Y90) transcatheter arterial radioembolization (TARE), she was deemed ineligible due to high intralesional shunting (approximately 27%) observed during planning angiography. To slow interval growth of the hepatic lesions and improve symptom control, she underwent transcatheter arterial embolization of the dominant 8 cm right hepatic mass using bland particles (>250 µm to accommodate the intralesional shunting). Embolization was performed to complete stasis, and the patient recovered without complications (**Figure 3**). Seven days later, she started combination immunotherapy again with ipilimumab (3 mg/kg) and nivolumab (1 mg/kg) to target her extrahepatic disease.

**Figure 4:**
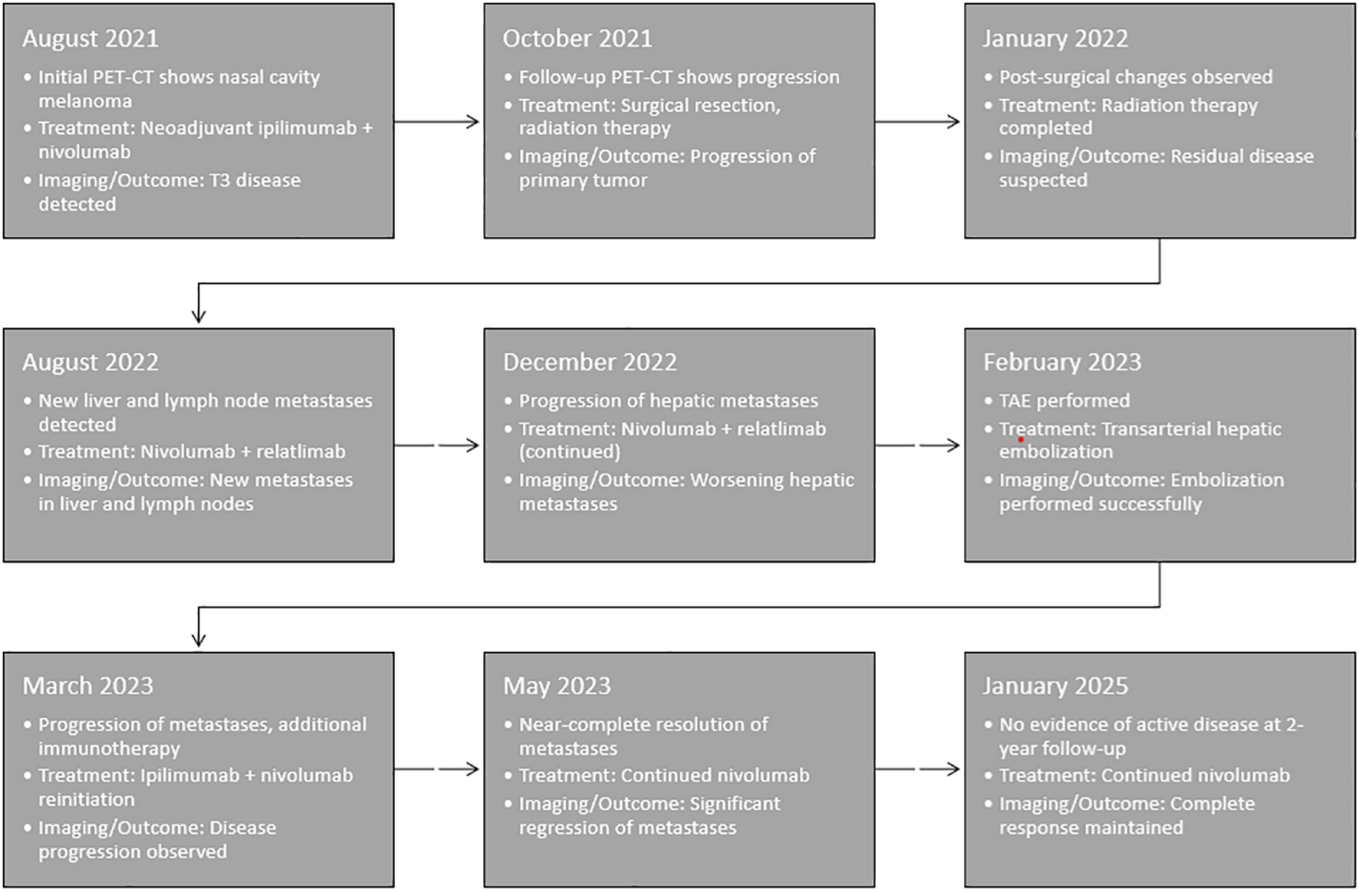
Timeline of key clinical events, including the administration of local therapies, sequential immunotherapy treatments, and the observed abscopal effect. This visual representation highlights the temporal relationship between transarterial hepatic embolization (TAE), immunotherapy reintroduction, and the subsequent systemic response.

Repeat PET-CT on March 14, 2023, after 2 cycles of immunotherapy, revealed progressive FDG-avid metastases in the liver, lungs, right pleura, retrocrural region, and multifocal osseous sites (6). The largest hepatic lesion in the right hepatic lobe exhibited areas of central necrosis and a maximum standardized uptake value (SUV) of 10.6. Post-treatment changes were noted in the low-attenuation areas, showing no activity. Additionally, FDG-avid peritoneal disease was identified, with the largest lesion measuring up to 2.9 cm with an SUV of 7.1 in the right upper abdominal quadrant. Despite the evidence of radiographic progression, the patient subjectively felt better, and with a lack of effective alternative systemic therapies, the patient received two additional cycles of ipilimumab and nivolumab in March and April 2023. On May 4, 2023, additional imaging including PET-CT and MRI of the orbits, were performed for staging, which demonstrated near-complete resolution of metastases in the lungs, pleura, lymph nodes, bones, and subcutaneous tissue. Few foci of residual activity remained in the liver, but those sites revealed overall improvement and without new lesion identified. MRI orbits also showed complete treatment response, with resolution of the right premaxillary soft tissue nodule and left dorsal T4-T5 paravertebral soft issues. A partial treatment response was also seen in the reduction of the right retropharyngeal adenopathy. The patient has subsequently continued single agent nivolumab immunotherapy. She has had a biopsy-proven recurrence in a single pelvic lymph node that subsequently responded with continued nivolumab and remains free of evidence of any active or progressing disease 24 months after liver embolization and immunotherapy re-challenge. To provide a clearer understanding of the sequence of treatments and their potential impact, we have included a timeline outlining key events in the patient’s course, including the administration of local therapies, immunotherapy treatments, and the observed abscopal effect (**Figure 4**)

## Discussion

We report the case of a patient with metastatic mucosal melanoma initially resistant to ipilimumab and nivolumab immunotherapy, who after transarterial hepatic embolization and reinitiation of ipilimumab and nivolumab experienced a dramatic abscopal effect with disease regression in virtually all metastatic sites. No prior cases have been reported of abscopal effect after transcatheter hepatic arterial embolization.

The abscopal effect refers to a phenomenon where localized treatment, typically radiation therapy, causes not only reduction in the treated tumor but also leads to regression of distant, untreated tumors. The effect is thought to be secondary to activation of the immune response to the tumor’s destruction. Tumor destruction in turn triggers an immune response that targets and eliminates cancer cells outside the treated area. The abscopal effect is a relatively rare clinical occurrence, with one study finding 23 case reports of abscopal effect after isolated radiotherapy (7). Notably, a recent case report by Cerbon et al. described an abscopal response in visceral and osseous metastases following stereotactic body radiotherapy (SBRT) in combination with nivolumab and relatlimab for sinonasal mucosal melanoma (8). This highlights the potential for immune checkpoint blockage to synergize local therapies to induce systemic anti-tumor effects.

The immune response following embolization of hepatic tumors has not been fully elucidated and remains an area of active investigation. The precise characteristics have been explored in prior studies, for example, patients treated with trans-arterial chemoembolization (TACE) for hepatocellular carcinoma (HCC) have been found to exhibit lower densities of CD4+/FOXP3+, CD8+, and CD8+/PD-1+ T-cells in the intratumoral area compared to untreated patients (9). Interestingly, no significant differences in T-cell distribution were noted in the peritumoral area, except for CD8+/PD-1+ cells in the non-tumoral background. These findings suggest that TACE induces a distinct phenotypic profile of T-cell infiltrates in the tumor, characterized by reduced densities of both cytotoxic and regulatory T-cells. This observation aligns with the notion that embolization therapies, such as TACE, may alter the immune landscape within the tumor, potentially influencing the tumor’s ability to escape immune surveillance.

Further studies have highlighted the role of immune cells in mediating distant tumor regression following localized treatment, such as radiation. Research in athymic mouse models suggests that T-cells are crucial for targeting distant metastases after localized radiation (10). Radiation-induced DNA damage in tumor cells triggers the release of tumor-associated antigens, proinflammatory cytokines, chemokines, danger signals, and upregulates various cell adhesion molecules and death receptors, including Fas and major histocompatibility complex (MHC) class I and II on tumor cells (11–13). These signals promote the recruitment of innate immune cells and tumor-specific lymphocytes to the irradiated site, while also enhancing dendritic cell (DC) activation in circulation. Additionally, radiation can enhance the antigen-presenting capabilities of DCs, which in turn can amplify the anti-tumor immune response. This series of immune events provide a strong rationale for combining radiation therapy with immunotherapies to potentiate anti-tumor effects, particularly through mechanisms like the abscopal effect, where distant, non-irradiated tumors also respond to treatment.

Abscopal effects require both effective antigen release and antigen-specific T cell activation. The optimal combination of local therapy and immune-activating therapy remains uncertain. In the present case, the patient had previously received remote external beam radiation and immunotherapy but experienced the abscopal effect only after undergoing transarterial hepatic embolization (TAE) and the reintroduction of immunotherapy. This timing suggests a more direct association between the abscopal effect and the combination of TAE with immunotherapy. It raises the question of whether cumulative tumor damage from sequential local therapies facilitated enhanced antigen release, while the use of three different immune checkpoint inhibitors contributed to melanoma-specific T cell activation. The sequential use of different ICIs, including nivolumab and relatlimab, may have helped overcome primary resistance mechanisms that initially limited response to neoadjuvant ipilimumab and nivolumab. Understanding how different local and systemic therapies interact to promote effective immune responses is crucial for optimizing future therapeutic strategies in mucosal melanoma.

Immune checkpoint inhibitors have significantly improved the prognosis of melanoma, yet melanoma cells have developed complex mechanisms to evade immune surveillance in the presence of ICIs. These mechanisms include alterations in melanoma cell immunogenicity, immune cell trafficking, T-cell priming, and immune cell exhaustion, as well as tumor neovascularization, modulation of the tumor microenvironment (TME), and metabolic antagonism within the tumor (14). Resistance to ICIs can also be driven by negative immune checkpoints and variations in the gut microbiota, among other factors (15). As a result, overcoming resistance to ICIs requires strategies that modulate the TME, enhance T-cell function, and address tumor heterogeneity. Combination therapies that target these multiple resistance mechanisms hold promise for improving outcomes in melanoma patients.

This case report provides a compelling anecdote of the potential benefit of combining local embolization therapy with immune checkpoint inhibition in metastatic mucosal melanoma. As noted previously, patients with mucosal melanoma have lower response rates to U.S. Food and Drug Administration-approved immunotherapies than patients with cutaneous melanoma (4). In this patient, hepatic embolization of an isolated tumor with concurrent ipilimumab and nivolumab resulted in the near-complete resolution of multiple distant metastases, including those in the lungs, pleura, lymph nodes, osseous structures, and subcutaneous tissues, despite previously failing to respond to this combination of agents in the neo-adjuvant setting. While a different dosing regimen (ipilimumab 3 mg/kg and nivolumab 1 mg/kg) was used to treat the patient after embolization than had been used in the neoadjuvant setting (ipilimumab 1 mg/kg and nivolumab 3 mg/kg), a prospective randomized clinical trial in metastatic melanoma patients showed no significant difference in progression-free survival or overall survival between these two regimens (16). This case also highlights the ability of this combination approach to overcome resistance to combination immunotherapy with ipilimumab and nivolumab. Notably, the marked response in this patient after significant progression after 2 cycles of treatment contrasts with results reported in the recent ADAPT-IT prospective clinical trial (6). In that trial, metastatic melanoma patients (n=60) underwent restaging after 2 cycles of treatment with ipilimumab (3 mg/kg) and nivolumab (1 mg/kg). Patients without evidence of disease progression (n=41) switched to single-agent nivolumab at that time, while patients with disease progression (n=19) received 2 additional cycles of combination immunotherapy, followed by single-agent nivolumab for up to 2 years among patients who achieved a response or stable disease. Among the progressing patients, ultimately the best response to ipilimumab and nivolumab was progressive disease (PD) in 13 (68.4%), stable disease (SD) in 4 (21.1%), and clinical response (ORR) in 2 (10.5%). Both responders in that trial had mixed responses with a single progressing tumor after 2 cycles of treatment, in contrast to the widespread progression observed in the patient described in this case report.

Despite the valuable clinical evidence this case report provides, there are limitations to the conclusions drawn from this single patient. These include limited generalizability and lack of evidence that this treatment approach would be effective for other patients with metastatic mucosal melanoma. The lack of biopsies to characterize the immune effects of embolization is also a limitation of this case report but supports the rationale for future studies to address this deficiency.

## Conclusion

In conclusion, this case underscores the therapeutic potential of integrating local embolization with immunotherapy in metastatic melanoma. This case proves not only the possibility of overcoming immunotherapy resistance but also the remarkable clinical benefit of the abscopal effect in treating distant metastases – even in patients with immunotherapy-resistant mucosal melanoma. Further studies are called for to explore the underlying immune mechanisms and optimize treatment strategies for patients with metastatic melanoma.

## Data Availability

The raw data supporting the conclusions of this article will be made available by the authors, without undue reservation.
